# Direct Identification of Functional Amyloid Proteins by Label-Free Quantitative Mass Spectrometry

**DOI:** 10.3390/biom7030058

**Published:** 2017-08-04

**Authors:** Heidi N. Danielsen, Susan H. Hansen, Florian-Alexander Herbst, Henrik Kjeldal, Allan Stensballe, Per H. Nielsen, Morten S. Dueholm

**Affiliations:** 1 Center for Microbial Communities, Department of Chemistry and Bioscience, Aalborg University, 9220 Aalborg, Denmark; nolsoe83@gmail.com (H.N.D.); shh@bio.aau.dk (S.H.H.); fah@bio.aau.dk (F.-A.H.); kjeldal@bio.aau.dk (H.K.); phn@bio.aau.dk (P.H.N.); 2 Department of Health Science and Technology, Aalborg University, 9220 Aalborg, Denmark; as@hst.aau.dk

**Keywords:** functional amyloids, biofilm, nanomaterials, mass spectrometry

## Abstract

Functional amyloids are important structural and functional components of many biofilms, yet our knowledge of these fascinating polymers is limited to a few examples for which the native amyloids have been isolated in pure form. Isolation of the functional amyloids from other cell components represents a major bottleneck in the search for new functional amyloid systems. Here we present a label-free quantitative mass spectrometry method that allows identification of amyloid proteins directly in cell lysates. The method takes advantage of the extreme structural stability and polymeric nature of functional amyloids and the ability of concentrated formic acid to depolymerize the amyloids. An automated data processing pipeline that provides a short list of amyloid protein candidates was developed based on an amyloid-specific sigmoidal abundance signature in samples treated with increasing concentrations of formic acid. The method was evaluated using the *Escherichia coli* curli and the *Pseudomonas* Fap system. It confidently identified the major amyloid subunit for both systems, as well as the minor subunit for the curli system. A few non-amyloid proteins also displayed the sigmoidal abundance signature. However, only one of these contained a sec-dependent signal peptide, which characterizes most of all secreted proteins, including all currently known functional bacterial amyloids.

## 1. Introduction

Amyloids are highly ordered protein fibrils defined by a cross-β-sheet quaternary structure and the ability to self-assemble from their monomeric counterparts in a nucleation-dependent process [1]. Many amyloids display exceptional resistance towards thermal and chemical denaturants due to a tightly packed cross-β structure [2]. Consequently, they are ideal structural materials in biological systems, and organisms from all domains of life exploit amyloids for beneficial purposes [3,4]. Functional amyloids have also shown promising results as tunable nanomaterials. The well-described *Escherichia coli* curli system was for example used to engineer biofilm properties by genetically attaching functional domains from other proteins to the major amyloid subunit (CsgA) [5]. Another type of amyloid that has shown potential applications as a nanomaterial is the hydrophobins expressed by fungi. Hydrophobins have a special ability to position themselves at water-air or solid-water interfaces, thus making them very applicable within protein purification. Furthermore, they show possibilities within the food industry, in pharmaceuticals, and for biotechnological processes [6]. With the numerous possible applications of amyloids, it is of great interest to discover new amyloid systems from a variety of different microorganisms.

Microbial biofilms represent bacteria embedded in an extracellular matrix (ECM) composed mainly of polysaccharides, extracellular DNA, and proteins [7]. The ECM enables the bacteria to organize spatially, which allows for functional differentiation [8,9]. It also provides protection against environmental and chemical stresses, including the action of antibiotics and host immune response. The biofilm lifestyle is accordingly favored by the majority of all bacteria [9], and biofilm formation by pathogenic bacteria is tightly associated with the development of chronic infections [10]. Previous studies have indicated that amyloids are common components of almost all microbial biofilms independent of habitat [11,12]. They are therefore believed to play essential roles in biofilm ecology [13]. However, our current knowledge of functional amyloids is restricted to a few amyloid systems, for which the amyloids have been purified and characterized in detail [4]. The identification and characterization of more amyloid systems will likely provide a deeper insight into the many roles of amyloids in biofilms.

Functional amyloid systems are traditionally discovered based on the isolation of native amyloids and the validation of the amyloid structure through biophysical characterization. The isolation of amyloids is unfortunately not a straightforward task. Insolubility and extreme stability, which characterize most functional amyloids, exclude them from standard protein analyses, including sodium dodecyl sulfate polyacrylamide gel electrophoresis (SDS-PAGE) and mass spectrometry-based characterization without special pretreatment. Furthermore, many functional amyloids are highly adhesive and therefore easily lost during purification due to binding to consumables such as pipet tips and sample tubes. Consequently, the current methods require pure cultures, which can express a large number of amyloids under laboratory conditions. A purification-independent technique for amyloid protein identification would provide the means to identify less abundant amyloids, as well as amyloids that are only expressed upon exposure to habitat-specific environmental cues, including stress conditions and host defense mechanisms [14].

We here present a quantitative proteomics technique that facilitates the direct identification of functional amyloid candidates in cell lysates. This technique is specific, sensitive, and provides an opportunity to identify amyloids in complex samples with low bacterial diversity, such as clinical biofilms.

## 2. Results

### 2.1. Description of the Method

**Figure 1:**
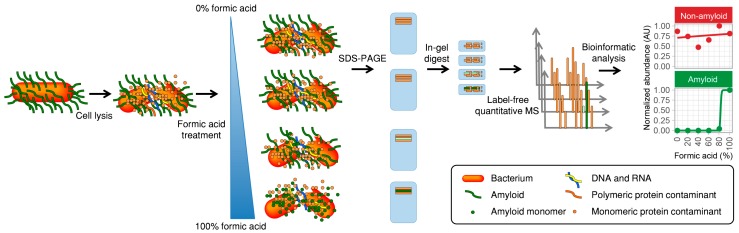
Direct identification of functional amyloid proteins using label-free quantitative (LFQ) Liquid chromatography–tandem mass spectrometry (LC-MS/MS). The sample is lysed and divided into aliquots that are lyophilized and treated with either 0%, 20%, 40%, 60%, 80%, or 100% formic acid. The samples are then lyophilized, dissolved in reducing sodium dodecyl sulfate polyacrylamide gel electrophoresis (SDS-PAGE) loading buffer, and subjected to short run SDS-PAGE. The amyloid proteins can only enter the gel if they have been pretreated with concentrated formic acid and are therefore only present in these samples. In-gel digestion is carried out with trypsin, and samples analyzed by label-free quantitative LC-MS/MS using MaxQuant and the MaxLFQ algorithm. The data is finally analyzed for each protein using an automated script, and positive amyloid candidates are identified based on their abundance profiles with respect to the formic acid concentration.

An overview of the new method for amyloid detection is presented in Figure 1. The bacteria in the sample are first lysed to release intracellular proteins. Next, aliquots of the total cell lysate are lyophilized and treated with formic acid at concentrations ranging from 0% to 100%. Many functional amyloids are only depolymerized in aggressive solvents such as concentrated formic acid [15,16], trifluoroacetic acid (TFA) [17,18], or hexafluoroisopropanol (HFIP) [19]. Functional amyloids are therefore only depolymerized in the samples treated with high concentrations of formic acid. The samples are lyophilized again to remove water and formic acid, dissolved in a special reducing SDS-PAGE loading buffer [20], and subjected to a brief run on an SDS-PAGE gel. The SDS-PAGE has two purposes: it removes cell debris and the native amyloids that are not able to enter the gel, and it retains the depolymerized amyloid proteins in an exposed monomeric conformation amenable to proteolytic digestion. The gel-embedded proteins are subjected to in-gel tryptic digest, and the resulting peptides are analyzed by mass spectrometry. Protein identification and quantification are done using MaxQuant [21] with the label-free quantification (LFQ) algorithm [22]. The LFQ values are normalized between individual measurements to prevent systematic errors such as deviations in sample loading, and are based on at least two quantifiable peptides. This allows the relative abundances of the same protein to be compared across samples. Amyloid proteins are suspected to show much higher abundance in samples treated with a higher concentration of formic acid, and they will therefore produce a characteristic sigmoidal abundance signature when the relative abundance is plotted against the formic acid concentration (Figure 1).

The mass spectrometry analysis results in the identification of thousands of proteins, and it is an enormous task to review the abundance signature for each protein. To ease this process, we created an R-markdown script that automatically identifies functional amyloid candidates based on their abundance signatures (Script S1). The script uses the raw MaxQuant data as input and separates the data for each protein. It then normalizes the abundance data according to the sample with the highest concentration giving it a value of 1. The normalized data for each protein is then fitted to a generalized linear model of the logistic function:

f(*x*) = 1/(1 + exp(−(a + b*x*))), (1)

The formic acid concentration where half of the amyloids have been depolymerized (f50) is:

f50 = −a/b,(2)

The slope of the fit at f50 is calculated as:

f’(f50) = b/4,(3)

We here define functional amyloids candidates as proteins that fulfill the following requirements:

(4) f50 > 60,f50 < 100,f′(f50) > 0.025

### 2.2. Method Validation with Cell Lysates of *E. coli* SM2258

**Figure 2:**
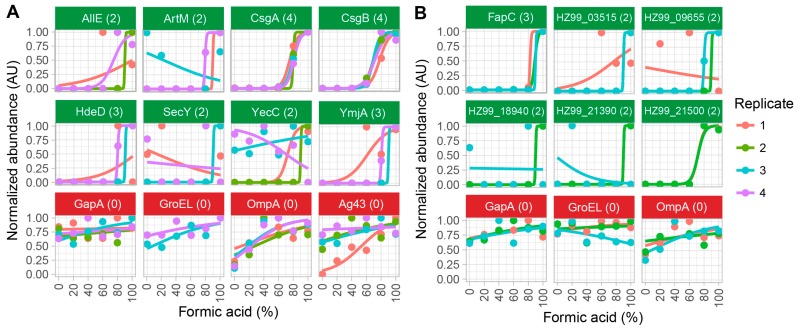
The identification of functional amyloid candidates in cell lysates of (**A**) *Escherichia coli* SM2258 and (**B**) *Pseudomonas* sp. UK4. Proteins with amyloid-specific abundance signatures in at least two biological replicates are shown with green titles. Negative controls are shown in red titles, which include the household proteins, Glyceraldehyde-3-phosphate dehydrogenase A (GapA) and 60 kDa chaperonin (GroEL); and β-barrel outer membrane proteins, like outer membrane protein A (OmpA) and antigen 43 (Ag43). The numbers in the parentheses indicate the number of biological replicates in which the proteins were classified as amyloid candidates based on the fitting parameters. Notice that some data points and curves are hidden as they overlap, and some proteins were not observed in all replicates.

The method was first evaluated with four biological replicates of the amyloid-producing *E. coli* strain SM2258 [11] grown at conditions known to promote curli expression (Figure 2A). The curli amyloids are composed of two subunits, the major subunit CsgA and the minor subunit CsgB [23].

Mass spectrometry analysis identified a total of 1438 proteins (Figure S1), of which 56 were classified as functional amyloid candidates (Table S1). However, many of the identified candidates were low abundant proteins that were only supported by data from a single biological replicate. To remove false positive hits, only proteins that were identified as amyloids in at least two of the replicates were considered. This reduced the list of amyloid candidates to eight (Figure 2). All currently known functional bacterial amyloids contain Sec-dependent signal peptides that mark them for secretion. The identified candidates were therefore analyzed for the presence of sec-dependent signal peptides using the SignalP 4.1 algorithm [24] (Table S1). Only two of the eight amyloid candidates contained Sec-dependent signal peptides, namely the major (CsgA) and the minor (CsgB) curli amyloid subunits. These two proteins were also the only proteins identified as amyloid candidates in all four biological replicates, demonstrating the specificity and sensitivity of the method.

### 2.3. Method Validation with Cell Lysates of *Pseudomonas* sp. UK4

To further evaluate the method, we analyzed three biological replicates of *Pseudomonas* sp. UK4 grown at conditions known to promote functional amyloids of *Pseudomonas* (Fap) expression [16] (Figure 2B). The Fap amyloids are composed of the major subunit FapC and the minor subunit FapB [16]. A third protein FapE has also been observed in the purified preparations of Fap fibrils, but it is uncertain if this protein is an integrated part of the amyloid [20]. *Pseudomonas* sp. UK4 produce significantly less amyloid compared to *E. coli* SM2258, and is likely a better model for other wild-type strains producing amyloids.

Mass spectrometry analysis identified a total of 2309 proteins (Figure S2), of which 46 were classified as functional amyloid candidates (Table S2). Only six of these were supported by data from at least two replicates. Two of these proteins contained Sec-dependent signal peptides according to SignalP 4.1 analysis. One was the major functional amyloid subunit (FapC), the other was a low abundant glutamine ABC transporter substrate-binding protein (HZ99_18940). Only FapC was identified in all three biological replicates. The minor amyloid subunit (FapB) was not detected in the mass spectrometry data due to low abundance. The confident detection of FapC as an amyloid protein, even though it was expressed at a significantly lower level than the *E. coli* curli, confirms the specificity and sensitivity of the method. 

## 3. Discussion

The isolation of functional amyloids from other cell components represents a major obstacle in the identification of new functional amyloid systems [4]. The method described here allows identification of functional amyloid proteins directly in cell lysates without any purification steps. It thus provides an opportunity to screen for amyloids in systems, where it is difficult or impossible to separate the native amyloids from contaminating cell components. By applying the method to two evolutionarily distinct amyloid systems, it was shown that the method is both sensitive and specific. The method returned only a few false positive amyloid candidates when biological replicates were considered, and the number of false positives could easily be reduced based on the presence or absence of Sec-dependent signal peptides, which characterizes all currently described functional bacterial amyloid proteins. However, cell lysis products are known to play important roles in biofilm structure and function, and it is possible the cytosolic proteins released may form amyloids when exposed to the extracellular environment. Such amyloids would be missed with the signal peptide criterion.

Another problem with the identification of novel functional amyloid systems relies on the fact that many bacteria require specific environmental stimuli to activate amyloid expression [25]. Such stimuli may involve interaction with host surface molecules, or they may be too complex to replicate in the laboratory. However, the current method may provide a solution to this problem. Due to the high sensitivity of the method, it may allow for the identification of amyloids directly in complex samples such as environmental biofilms or in cocultures of bacteria and their hosts.

We have previously hypothesized that many biofilm-forming pathogenic bacteria may use amyloid proteins during infections [26,27]. Now we can test this hypothesis by applying the current method to samples obtained from chronic wounds of immune-impaired individuals or sputum samples from patients suffering from cystic fibrosis. If amyloids are identified as important virulence factors, they may be targeted with specific or more general inhibitors, represented by CsgC for *E. coli* [28] and epigallocatechin gallate (EGCG) for *Pseudomonas* Fap [29], respectively.

It should be stressed that the method itself cannot be used to confirm whether a protein is an amyloid component. This still requires biophysical characterization of the native amyloid fibrils. However, with the current method and access to genomes or the relevant strains, we can identify the operons encoding the amyloids. These operons may then be expressed in bacteria that allow purification of the native amyloids, as has previously been done for the *fap*-operon from various species of *Pseudomonas* [16,20]. Another limitation of the method is that it can only be used to identify extremely stable amyloids that require more 50% formic acid to be depolymerized. The TasA amyloids from *Bacillus subtilis* are known to be depolymerized already at 20% formic acid [30]. The method will therefore not identify TasA as an amyloid protein. The current approach also misses proteins that occur both in a soluble and an amyloid form within the same sample, e.g., the phenol soluble modulins of *Staphylococcus aureus*. However, despite these limitations, we believe that the current method will provide a useful tool for many working within the field of functional amyloids, and that it will expedite the identification of future functional amyloid systems.

## 4. Materials and Methods

### 4.1. Bacterial Cultures and Activated Sludge

Glycerol stocks of *E. coli* MG1665 str. SM2258 [11] and *Pseudomonas* sp. UK4 [16,31] were used to inoculate 10 mL of colonization factor antigen (CFA) medium (10 g/L hydrolyzed casein, 50 mg/L MgSO4, 5 mg/L MnCl2, 1.5 g/L yeast extract, pH 7.4) in 50-mL Greiner tubes. The bacteria were grown overnight at 26 °C with 150 rpm of agitation in an Innova 40 Benchtop Incubator Shaker (Eppendorf, Hamburg, Germany). Then, 100 µL aliquots of each overnight culture were spread on 10 CFA agar plates solidified with 2% agar, which were incubated at 26 °C for 72 h. Bacteria from 10 plates were scraped off and resuspended in 10 mL buffer (10 mM Tris-HCl, pH 8.0).

### 4.2. Sample Preparation for Mass Spectrometry

For sample preparation, 4 × 1 mL of bacterial culture were transferred to lysing matrix E tubes (MP Biomedicals, Eschwege, Germany). Subsequently, 10 µL of Halt protease and phosphatase inhibitor cocktail (ThermoFisher Scientific, Waltham, MA, USA) were added to each tube to prevent protein degradation during cell lysis. The samples were then lysed by bead beating in a FastPrep-24 instrument for 3 × 20 s at 6.0 m/s. The samples were incubated on ice for 2 min between each bead beating to prevent thermally induced protein denaturation. Beads were allowed to settle by gravity for 5 min, after which 500 µL cell lysate were collected from the four tubes and combined. Then, 50 µL aliquots of the cell lysate were transferred to six Eppendorf tubes in three or four replicates. The replicates were lyophilized and resuspended in 100 µL of either 0%, 20%, 40%, 60%, 80% or 100% formic acid. The samples were lyophilized again and then resuspended in 100 µL of reducing SDS-PAGE loading buffer containing 8 M of urea [20]. Insoluble material was pelleted by centrifugation for 1 min at 22,000× *g*, and 15 µL of supernatant was loaded on 12% SDS-PAGE gels. Electrophoresis was carried out at 140 V for 5 min, and the gels were stained with Coomassie Brilliant Blue G250 (ThermoFisher Scientific). The narrow bands containing all proteins were excised and subjected to tryptic in-gel digestion [32]. Tryptic peptides were reconstituted in 5% formic acid and purified using StageTips packed with PorosOligo R3 material (Applied Biosystems, Foster City, CA, USA) on top of two C18 disks (3 M, Bioanalytical Technologies, St. Paul, MN. USA) as previously described [33,34]. Peptides were eluted with 66% (*v*/*v*) acetonitrile (ACN) and dried by vacuum centrifugation without heating.

### 4.3. Mass Spectrometry

Ultra-performance liquid chromatography (UPLC) tandem mass spectrometry analysis was performed on an ultimate 300 UPLC system (ThermoFisher Scientific) coupled online to a Q Exactive Plus mass spectrometer (ThermoFisher Scientific). Desalted peptides were reconstituted in 0.1% trifluoroacetic acid and 2% acetonitrile. Of each sample, 8 µL were injected by the autosampler and concentrated on a trapping column (Pepmap100, C18, 100 μm × 2 cm, 5 μm, ThermoFisher Scientific) with water containing 0.1% formic acid and 2% ACN at flow rates of 4 μL min^−1^. After 5 min, the peptides were loaded onto a separation column (PepmapRSLC, C18, 75 μm i.d. × 75 cm, 100 Å, ThermoFisher Scientific). Chromatography was performed with 0.1% formic acid in solvent A (100% water) and B (100% acetonitrile) using a ramp gradient. The concentration of B was increased from 2% to 8% over 1 min, followed by an increase from 8% to 30% over 39 min. Solvent B was subsequently increased from 30% to 90% within 5 min and maintained at this level for 3 min.

The mass spectrometry proteomics data was deposited to the ProteomeXchange Consortium via the PRIDE [35] partner repository with the dataset identifier PXD006835.

### 4.4. Data Analysis

Protein identification and quantification were done with the open-source software MaxQuant v1.5.8.3 [21]. The sequence databases for *E. coli* str. K-12 MG1655 and *Pseudomonas* sp. UK4 were retrieved from the National Center for Biotechnology Information (NCBI) (tax: 511145 and 452680) [36]. Besides the standard settings, LFQ [22] was activated in MaxQuant. This included a peptide and protein false discovery rate of 1%. Reversed sequences as decoys and contaminant sequences were added automatically by MaxQuant. The minimum ratio count for LFQ was set to one. Complete lists of the protein and peptide identifications, as well as detailed parameters, are available together with the deposited mass spectrometric data. The reverse and contaminant sequences were removed from the MaxQuant output, and unique identifiers (gene names or id numbers) were created for each protein. The resulting dataset was loaded into R and analyzed using an automated R-markdown script (Script S1).

## Supplementary Materials

The following are available online at www.mdpi.com/2218-273X/7/3/58/s1, Figure S1: Abundance profiles for all proteins in the *E. coli* SM2258 sample, Figure S2: Abundance profiles for all proteins in the *Pseudomonas* sp. UK4 sample, Table S1: Amyloid candidates identified in the *E. coli* SM2258 sample, Table S2: Amyloid candidates identified in the *Pseudomonas* sp. UK4 sample, Script S1: R-markdown script used for automated identification of amyloid protein candidates.
